# Supplementary material for: Comparative Analysis of Proteomes of a Number of Nosocomial Pathogens by KEGG Modules and KEGG Pathways
Source: Int J Mol Sci. 2020 Oct 22;21(21):7839. doi: 10.3390/ijms21217839 (PMC7660090; doi:10.3390/ijms21217839)
Supplement: Supplementary file 1 [file ijms-21-07839-s001.pdf]

Supplementary Table S1. *S. aureus* KEGG-modules.

| KEGG-module ID | KEGG-module description | Genomes | Frequency |
|---|---|---|---|
| **M00001** | Glycolysis (Embden-Meyerhof pathway), glucose => pyruvate | 53 | 1 |
| **M00002** | Glycolysis, core module involving three-carbon compounds | 53 | 1 |
| **M00007** | Pentose phosphate pathway, non-oxidative phase, fructose 6P => ribose 5P | 53 | 1 |
| **M00010** | Citrate cycle, first carbon oxidation, oxaloacetate => 2-oxoglutarate | 53 | 1 |
| **M00018** | Threonine biosynthesis, aspartate => homoserine => threonine | 53 | 1 |
| **M00022** | Shikimate pathway, phosphoenolpyruvate + erythrose-4P => chorismate | 53 | 1 |
| **M00045** | Histidine degradation, histidine => N-formiminoglutamate => glutamate | 53 | 1 |
| **M00050** | Guanine ribonucleotide biosynthesis IMP => GDP,GTP | 53 | 1 |
| **M00052** | Pyrimidine ribonucleotide biosynthesis, UMP => UDP/UTP,CDP/CTP | 53 | 1 |
| **M00082** | Fatty acid biosynthesis, initiation | 53 | 1 |
| **M00120** | Coenzyme A biosynthesis, pantothenate => CoA | 53 | 1 |
| **M00123** | Biotin biosynthesis, pimeloyl-ACP/CoA => biotin | 53 | 1 |
| **M00140** | C1-unit interconversion, prokaryotes | 53 | 1 |
| **M00307** | Pyruvate oxidation, pyruvate => acetyl-CoA | 53 | 1 |
| **M00345** | Formaldehyde assimilation, ribulose monophosphate pathway | 53 | 1 |
| **M00364** | C10-C20 isoprenoid biosynthesis, bacteria | 53 | 1 |
| **M00549** | Nucleotide sugar biosynthesis, glucose => UDP-glucose | 53 | 1 |
| **M00579** | Phosphate acetyltransferase-acetate kinase pathway, acetyl-CoA => acetate | 53 | 1 |
| **M00580** | Pentose phosphate pathway, archaea, fructose 6P => ribose 5P | 53 | 1 |
| **M00700** | Multidrug resistance, efflux pump AbcA | 53 | 1 |
| **M00702** | Multidrug resistance, efflux pump NorB | 53 | 1 |
| **M00726** | Cationic antimicrobial peptide (CAMP) resistance, lysyl-phosphatidylglycerol (L-PG) synthase MprF | 53 | 1 |
| **M00730** | Cationic antimicrobial peptide (CAMP) resistance, VraFG transporter | 53 | 1 |
| **M00844** | Arginine biosynthesis, ornithine => arginine | 53 | 1 |
| **M00875** | Staphyloferrin B biosynthesis, L-serine => staphyloferrin B | 53 | 1 |

Supplementary Table S1 (continuation). *S. aureus* KEGG-modules.

| KEGG-module ID | KEGG-module description | Genomes | Frequency |
|---|---|---|---|
| **M00876** | Staphyloferrin A biosynthesis, L-ornithine => staphyloferrin A | 53 | 1 |
| **M00003** | Gluconeogenesis, oxaloacetate => fructose-6P | 52 | 0.98 |
| **M00009** | Citrate cycle (TCA cycle, Krebs cycle) | 52 | 0.98 |
| **M00011** | Citrate cycle, second carbon oxidation, 2-oxoglutarate => oxaloacetate | 52 | 0.98 |
| **M00021** | Cysteine biosynthesis, serine => cysteine | 52 | 0.98 |
| **M00049** | Adenine ribonucleotide biosynthesis, IMP => ADP,ATP | 52 | 0.98 |
| **M00086** | beta-Oxidation, acyl-CoA synthesis | 52 | 0.98 |
| **M00432** | Leucine biosynthesis, 2-oxoisovalerate => 2-oxoisocaproate | 52 | 0.98 |
| **M00555** | Betaine biosynthesis, choline => betaine | 52 | 0.98 |
| **M00577** | Biotin biosynthesis, BioW pathway, pimelate => pimeloyl-CoA => biotin | 52 | 0.98 |
| **M00005** | PRPP biosynthesis, ribose 5P => PRPP | 51 | 0.96 |
| **M00119** | Pantothenate biosynthesis, valine/L-aspartate => pantothenate | 45 | 0.85 |
| **M00157** | F-type ATPase, prokaryotes and chloroplasts | 45 | 0.85 |
| **M00416** | Cytochrome aa3-600 menaquinol oxidase | 45 | 0.85 |
| **M00704** | Tetracycline resistance, efflux pump Tet38 | 45 | 0.85 |
| **M00705** | Multidrug resistance, efflux pump MepA | 45 | 0.85 |
| **M00725** | Cationic antimicrobial peptide (CAMP) resistance, dltABCD operon | 45 | 0.85 |
| **M00083** | Fatty acid biosynthesis, elongation | 44 | 0.83 |
| **M00125** | Riboflavin biosynthesis, GTP => riboflavin/FMN/FAD | 44 | 0.83 |
| **M00530** | Dissimilatory nitrate reduction, nitrate => ammonia | 43 | 0.81 |
| **M00023** | Tryptophan biosynthesis, chorismate => tryptophan | 42 | 0.79 |
| **M00126** | Tetrahydrofolate biosynthesis, GTP => THF | 42 | 0.79 |
| **M00048** | Inosine monophosphate biosynthesis, PRPP + glutamine => IMP | 41 | 0.77 |
| **M00019** | Valine/isoleucine biosynthesis, pyruvate => valine / 2-oxobutanoate => isoleucine | 38 | 0.72 |
| **M00570** | Isoleucine biosynthesis, threonine => 2-oxobutanoate => isoleucine | 38 | 0.72 |
| **M00627** | beta-Lactam resistance, Bla system | 24 | 0.45 |
| **M00625** | Methicillin resistance | 13 | 0.25 |
| **M00714** | Multidrug resistance, efflux pump QacA | 11 | 0.21 |

Supplementary Table S2. *S. aureus* KEGG-pathways.

| KEGG-pathway ID | KEGG-pathway description | Genomes | Frequency |
|---|---|---|---|
| **00010** | Glycolysis / Gluconeogenesis | 53 | 1 |
| **00020** | Citrate cycle (TCA cycle) | 53 | 1 |
| **00030** | Pentose phosphate pathway | 53 | 1 |
| **00040** | Pentose and glucuronate interconversions | 53 | 1 |
| **00051** | Fructose and mannose metabolism | 53 | 1 |
| **00052** | Galactose metabolism | 53 | 1 |
| **00053** | Ascorbate and aldarate metabolism | 53 | 1 |
| **00061** | Fatty acid biosynthesis | 53 | 1 |
| **00071** | Fatty acid degradation | 53 | 1 |
| **00072** | Synthesis and degradation of ketone bodies | 53 | 1 |
| **00121** | Secondary bile acid biosynthesis | 53 | 1 |
| **00130** | Ubiquinone and other terpenoid-quinone biosynthesis | 53 | 1 |
| **00190** | Oxidative phosphorylation | 53 | 1 |
| **00220** | Arginine biosynthesis | 53 | 1 |
| **00230** | Purine metabolism | 53 | 1 |
| **00240** | Pyrimidine metabolism | 53 | 1 |
| **00250** | Alanine, aspartate and glutamate metabolism | 53 | 1 |
| **00260** | D-Alanine metabolism | 53 | 1 |
| **00261** | Monobactam biosynthesis | 53 | 1 |
| **00270** | Cysteine and methionine metabolism | 53 | 1 |
| **00280** | Valine, leucine and isoleucine degradation | 53 | 1 |
| **00281** | Geraniol degradation | 53 | 1 |
| **00290** | Valine, leucine and isoleucine biosynthesis | 53 | 1 |
| **00300** | Lysine biosynthesis | 53 | 1 |
| **00310** | Lysine degradation | 53 | 1 |
| **00330** | Arginine and proline metabolism | 53 | 1 |
| **00340** | Histidine metabolism | 53 | 1 |
| **00350** | Tyrosine metabolism | 53 | 1 |
| **00360** | Phenylalanine metabolism | 53 | 1 |
| **00362** | Benzoate degradation | 53 | 1 |
| **00380** | Tryptophan metabolism | 53 | 1 |
| **00400** | Phenylalanine, tyrosine and tryptophan biosynthesis | 53 | 1 |
| **00401** | Novobiocin biosynthesis | 53 | 1 |
| **00410** | beta-Alanine metabolism | 53 | 1 |
| **00430** | Taurine and hypotaurine metabolism | 53 | 1 |
| **00440** | Phosphonate and phosphinate metabolism | 53 | 1 |
| **00450** | Selenocompound metabolism | 53 | 1 |
| **00460** | Cyanoamino acid metabolism | 53 | 1 |
| **00471** | D-Glutamine and D-glutamate metabolism | 53 | 1 |
| **00472** | D-Arginine and D-ornithine metabolism | 53 | 1 |

Supplementary Table S2 (continuation). *S. aureus* KEGG-pathways.

| KEGG-pathway ID | KEGG-pathway description | Genomes | Frequency |
|---|---|---|---|
| **00473** | D-Alanine metabolism | 53 | 1 |
| **00480** | Glutathione metabolism | 53 | 1 |
| **00500** | Starch and sucrose metabolism | 53 | 1 |
| **00520** | Amino sugar and nucleotide sugar metabolism | 53 | 1 |
| **00521** | Streptomycin biosynthesis | 53 | 1 |
| **00550** | Peptidoglycan biosynthesis | 53 | 1 |
| **00561** | Glycerolipid metabolism | 53 | 1 |
| **00562** | Inositol phosphate metabolism | 53 | 1 |
| **00564** | Glycerophospholipid metabolism | 53 | 1 |
| **00590** | Arachidonic acid metabolism | 53 | 1 |
| **00592** | alpha-Linolenic acid metabolism | 53 | 1 |
| **00620** | Pyruvate metabolism | 53 | 1 |
| **00622** | Xylene degradation | 53 | 1 |
| **00625** | Chloroalkane and chloroalkene degradation | 53 | 1 |
| **00626** | Naphthalene degradation | 53 | 1 |
| **00630** | Glyoxylate and dicarboxylate metabolism | 53 | 1 |
| **00640** | Propanoate metabolism | 53 | 1 |
| **00650** | Butanoate metabolism | 53 | 1 |
| **00660** | C5-Branched dibasic acid metabolism | 53 | 1 |
| **00670** | One carbon pool by folate | 53 | 1 |
| **00680** | Methane metabolism | 53 | 1 |
| **00730** | Thiamine metabolism | 53 | 1 |
| **00740** | Riboflavin metabolism | 53 | 1 |
| **00750** | Vitamin B6 metabolism | 53 | 1 |
| **00760** | Nicotinate and nicotinamide metabolism | 53 | 1 |
| **00770** | Pantothenate and CoA biosynthesis | 53 | 1 |
| **00780** | Biotin metabolism | 53 | 1 |
| **00785** | Lipoic acid metabolism | 53 | 1 |
| **00790** | Folate biosynthesis | 53 | 1 |
| **00860** | Porphyrin and chlorophyll metabolism | 53 | 1 |
| **00900** | Terpenoid backbone biosynthesis | 53 | 1 |
| **00906** | Carotenoid biosynthesis | 53 | 1 |
| **00910** | Nitrogen metabolism | 53 | 1 |
| **00920** | Sulfur metabolism | 53 | 1 |
| **00970** | Aminoacyl-tRNA biosynthesis | 53 | 1 |
| **00998** | Biosynthesis of secondary metabolites - other antibiotics | 53 | 1 |
| **00999** | Biosynthesis of secondary metabolites - unclassified | 53 | 1 |
| **01100** | Metabolic pathways | 53 | 1 |
| **01110** | Biosynthesis of secondary metabolites | 53 | 1 |
| **01120** | Microbial metabolism in diverse environments | 53 | 1 |

Supplementary Table S2 (continuation). *S. aureus* KEGG-pathways.

| KEGG-pathway ID | KEGG-pathway description | Genomes | Frequency |
|---|---|---|---|
| **01130** | Biosynthesis of antibiotics | 53 | 1 |
| **01200** | Carbon metabolism | 53 | 1 |
| **01210** | 2-Oxocarboxylic acid metabolism | 53 | 1 |
| **01212** | Fatty acid metabolism | 53 | 1 |
| **01220** | Degradation of aromatic compounds | 53 | 1 |
| **01230** | Biosynthesis of amino acids | 53 | 1 |
| **01501** | beta-Lactam resistance | 53 | 1 |
| **01502** | Vancomycin resistance | 53 | 1 |
| **01503** | Cationic antimicrobial peptide (CAMP) resistance | 53 | 1 |
| **02010** | ABC transporters | 53 | 1 |
| **02020** | Two-component system | 53 | 1 |
| **02024** | Quorum sensing | 53 | 1 |
| **02060** | Phosphotransferase system (PTS) | 53 | 1 |
| **03010** | Ribosome | 53 | 1 |
| **03018** | RNA degradation | 53 | 1 |
| **03020** | RNA polymerase | 53 | 1 |
| **03030** | DNA replication | 53 | 1 |
| **03060** | Protein export | 53 | 1 |
| **03070** | Bacterial secretion system | 53 | 1 |
| **03410** | Base excision repair | 53 | 1 |
| **03420** | Nucleotide excision repair | 53 | 1 |
| **03430** | Mismatch repair | 53 | 1 |
| **03440** | Homologous recombination | 53 | 1 |
| **04122** | Sulfur relay system | 53 | 1 |
| **05150** | Staphylococcus aureus infection | 53 | 1 |
| **05100** | Bacterial invasion of epithelial cells | 37 | 0.70 |
| **00643** | Styrene degradation | 2 | 0.04 |
| **00627** | Aminobenzoate degradation | 1 | 0.02 |

Supplementary Table S3. *Enterobacter spp.* KEGG-modules.

| KEGG-module ID | KEGG-module description | Genomes | Frequency |
|---|---|---|---|
| **M00001** | Glycolysis (Embden-Meyerhof pathway), glucose => pyruvate | 35 | 1 |
| **M00002** | Glycolysis, core module involving three-carbon compounds | 35 | 1 |
| **M00003** | Gluconeogenesis, oxaloacetate => fructose-6P | 35 | 1 |
| **M00004** | Pentose phosphate pathway (Pentose phosphate cycle) | 35 | 1 |
| **M00005** | PRPP biosynthesis, ribose 5P => PRPP | 35 | 1 |
| **M00006** | Pentose phosphate pathway, oxidative phase, glucose 6P => ribulose 5P | 35 | 1 |
| **M00007** | Pentose phosphate pathway, non-oxidative phase, fructose 6P => ribose 5P | 35 | 1 |
| **M00008** | Entner-Doudoroff pathway, glucose-6P => glyceraldehyde-3P + pyruvate | 35 | 1 |
| **M00010** | Citrate cycle, first carbon oxidation, oxaloacetate => 2-oxoglutarate | 35 | 1 |
| **M00012** | Glyoxylate cycle | 35 | 1 |
| **M00015** | Proline biosynthesis, glutamate => proline | 35 | 1 |
| **M00016** | Lysine biosynthesis, succinyl-DAP pathway, aspartate => lysine | 35 | 1 |
| **M00018** | Threonine biosynthesis, aspartate => homoserine => threonine | 35 | 1 |
| **M00019** | Valine/isoleucine biosynthesis, pyruvate => valine / 2-oxobutanoate => isoleucine | 35 | 1 |
| **M00020** | Serine biosynthesis, glycerate-3P => serine | 35 | 1 |
| **M00021** | Cysteine biosynthesis, serine => cysteine | 35 | 1 |
| **M00023** | Tryptophan biosynthesis, chorismate => tryptophan | 35 | 1 |
| **M00028** | Ornithine biosynthesis, glutamate => ornithine | 35 | 1 |
| **M00051** | Uridine monophosphate biosynthesis, glutamine (+ PRPP) => UMP | 35 | 1 |
| **M00061** | D-Glucuronate degradation, D-glucuronate => pyruvate + D-glyceraldehyde 3P | 35 | 1 |
| **M00087** | beta-Oxidation | 35 | 1 |
| **M00120** | Coenzyme A biosynthesis, pantothenate => CoA | 35 | 1 |
| **M00125** | Riboflavin biosynthesis, GTP => riboflavin/FMN/FAD | 35 | 1 |
| **M00157** | F-type ATPase, prokaryotes and chloroplasts | 35 | 1 |
| **M00168** | CAM (Crassulacean acid metabolism), dark | 35 | 1 |
| **M00176** | Assimilatory sulfate reduction, sulfate => H2S | 35 | 1 |
| **M00307** | Pyruvate oxidation, pyruvate => acetyl-CoA | 35 | 1 |
| **M00432** | Leucine biosynthesis, 2-oxoisovalerate => 2-oxoisocaproate | 35 | 1 |
| **M00549** | Nucleotide sugar biosynthesis, glucose => UDP-glucose | 35 | 1 |

Supplementary Table S3 (continuation). *Enterobacter spp.* KEGG-modules.

| KEGG-module ID | KEGG-module description | Genomes | Frequency |
|---|---|---|---|
| **M00570** | Isoleucine biosynthesis, threonine => 2-oxobutanoate => isoleucine | 35 | 1 |
| **M00579** | Phosphate acetyltransferase-acetate kinase pathway, acetyl-CoA => acetate | 35 | 1 |
| **M00844** | Arginine biosynthesis, ornithine => arginine | 35 | 1 |
| **M00854** | Glycogen biosynthesis, glucose-1P => glycogen/starch | 35 | 1 |
| **M00855** | Glycogen degradation, glycogen => glucose-6P | 35 | 1 |
| **M00009** | Citrate cycle (TCA cycle, Krebs cycle) | 34 | 0.97 |
| **M00011** | Citrate cycle, second carbon oxidation, 2-oxoglutarate => oxaloacetate | 34 | 0.97 |
| **M00017** | Methionine biosynthesis, apartate => homoserine => methionine | 34 | 0.97 |
| **M00022** | Shikimate pathway, phosphoenolpyruvate + erythrose-4P => chorismate | 34 | 0.97 |
| **M00026** | Histidine biosynthesis, PRPP => histidine | 34 | 0.97 |
| **M00049** | Adenine ribonucleotide biosynthesis, IMP => ADP,ATP | 34 | 0.97 |
| **M00050** | Guanine ribonucleotide biosynthesis IMP => GDP,GTP | 34 | 0.97 |
| **M00052** | Pyrimidine ribonucleotide biosynthesis, UMP => UDP/UTP,CDP/CTP | 34 | 0.97 |
| **M00083** | Fatty acid biosynthesis, elongation | 34 | 0.97 |
| **M00086** | beta-Oxidation, acyl-CoA synthesis | 34 | 0.97 |
| **M00116** | Menaquinone biosynthesis, chorismate => menaquinol | 34 | 0.97 |
| **M00118** | Glutathione biosynthesis, glutamate => glutathione | 34 | 0.97 |
| **M00119** | Pantothenate biosynthesis, valine/L-aspartate => pantothenate | 34 | 0.97 |
| **M00123** | Biotin biosynthesis, pimeloyl-ACP/CoA => biotin | 34 | 0.97 |
| **M00126** | Tetrahydrofolate biosynthesis, GTP => THF | 34 | 0.97 |
| **M00127** | Thiamine biosynthesis, AIR => thiamine-P/thiamine-2P | 34 | 0.97 |
| **M00144** | NADH:quinone oxidoreductase, prokaryotes | 34 | 0.97 |
| **M00149** | Succinate dehydrogenase, prokaryotes | 34 | 0.97 |
| **M00150** | Fumarate reductase, prokaryotes | 34 | 0.97 |
| **M00530** | Dissimilatory nitrate reduction, nitrate => ammonia | 34 | 0.97 |
| **M00554** | Nucleotide sugar biosynthesis, galactose => UDP-galactose | 34 | 0.97 |
| **M00616** | Sulfate-sulfur assimilation | 34 | 0.97 |
| **M00632** | Galactose degradation, Leloir pathway, galactose => alpha-D-glucose-1P | 34 | 0.97 |
| **M00024** | Phenylalanine biosynthesis, chorismate => phenylalanine | 33 | 0.94 |
| **M00025** | Tyrosine biosynthesis, chorismate => tyrosine | 33 | 0.94 |
| **M00048** | Inosine monophosphate biosynthesis, PRPP + glutamine => IMP | 33 | 0.94 |

Supplementary Table S3 (continuation). *Enterobacter spp.* KEGG-modules.

| KEGG-module ID | KEGG-module description | Genomes | Frequency |
|---|---|---|---|
| **M00053** | Pyrimidine deoxyribonuleotide biosynthesis, CDP/CTP => dCDP/dCTP,dTDP/dTTP | 33 | 0.94 |
| **M00063** | CMP-KDO biosynthesis | 33 | 0.94 |
| **M00064** | ADP-L-glycero-D-manno-heptose biosynthesis | 33 | 0.94 |
| **M00082** | Fatty acid biosynthesis, initiation | 33 | 0.94 |
| **M00093** | Phosphatidylethanolamine (PE) biosynthesis, PA => PS => PE | 33 | 0.94 |
| **M00115** | NAD biosynthesis, aspartate => NAD | 33 | 0.94 |
| **M00117** | Ubiquinone biosynthesis, prokaryotes, chorismate => ubiquinone | 33 | 0.94 |
| **M00124** | Pyridoxal biosynthesis, erythrose-4P => pyridoxal-5P | 33 | 0.94 |
| **M00364** | C10-C20 isoprenoid biosynthesis, bacteria | 33 | 0.94 |
| **M00417** | Cytochrome o ubiquinol oxidase | 33 | 0.94 |
| **M00572** | Pimeloyl-ACP biosynthesis, BioC-BioH pathway, malonyl-ACP => pimeloyl-ACP | 33 | 0.94 |
| **M00034** | Methionine salvage pathway | 32 | 0.91 |
| **M00060** | KDO2-lipid A biosynthesis, Raetz pathway, LpxL-LpxM type | 32 | 0.91 |
| **M00096** | C5 isoprenoid biosynthesis, non-mevalonate pathway | 32 | 0.91 |
| **M00121** | Heme biosynthesis, plants and bacteria, glutamate => heme | 32 | 0.91 |
| **M00846** | Siroheme biosynthesis, glutamate => siroheme | 32 | 0.91 |
| **M00045** | Histidine degradation, histidine => N-formiminoglutamate => glutamate | 31 | 0.89 |
| **M00133** | Polyamine biosynthesis, arginine => agmatine => putrescine => spermidine | 31 | 0.89 |
| **M00338** | Cysteine biosynthesis, homocysteine + serine => cysteine | 31 | 0.89 |
| **M00631** | D-Galacturonate degradation (bacteria), D-galacturonate => pyruvate + D-glyceraldehyde 3P | 31 | 0.89 |
| **M00533** | Homoprotocatechuate degradation, homoprotocatechuate => 2-oxohept-3-enedioate | 31 | 0.89 |
| **M00550** | Ascorbate degradation, ascorbate => D-xylulose-5P | 30 | 0.86 |
| **M00153** | Cytochrome bd ubiquinol oxidase | 29 | 0.83 |
| **M00555** | Betaine biosynthesis, choline => betaine | 28 | 0.80 |
| **M00761** | Undecaprenylphosphate alpha-L-Ara4N biosynthesis, UDP-GlcA => undecaprenyl phosphate alpha-L-Ara4N | 27 | 0.77 |
| **M00696** | Multidrug resistance, efflux pump AcrEF-TolC | 26 | 0.74 |
| **M00565** | Trehalose biosynthesis, D-glucose 1P => trehalose | 26 | 0.74 |
| **M00552** | D-galactonate degradation, De Ley-Doudoroff pathway, D-galactonate => glycerate-3P | 25 | 0.71 |
| **M00793** | dTDP-L-rhamnose biosynthesis | 19 | 0.54 |

Supplementary Table S3 (continuation). *Enterobacter spp.* KEGG-modules.

| KEGG-module ID | KEGG-module description | Genomes | Frequency |
|---|---|---|---|
| **M00136** | GABA biosynthesis, prokaryotes, putrescine => GABA | 16 | 0.46 |
| **M00851** | Carbapenem resistance | 11 | 0.31 |
| **M00122** | Cobalamin biosynthesis, cobinamide => cobalamin | 7 | 0.20 |
| **M00568** | Catechol ortho-cleavage, catechol => 3-oxoadipate | 5 | 0.14 |
| **M00551** | Benzoate degradation, benzoate => catechol / methylbenzoate => methylcatechol | 4 | 0.11 |
| **M00035** | Methionine degradation | 3 | 0.09 |
| **M00134** | Polyamine biosynthesis, arginine => ornithine => putrescine | 3 | 0.09 |
| **M00175** | Nitrogen fixation, nitrogen => ammonia | 3 | 0.09 |
| **M00545** | Trans-cinnamate degradation, trans-cinnamate => acetyl-CoA | 2 | 0.06 |
| **M00345** | Formaldehyde assimilation, ribulose monophosphate pathway | 2 | 0.06 |
| **M00580** | Pentose phosphate pathway, archaea, fructose 6P => ribose 5P | 2 | 0.06 |
| **M00046** | Pyrimidine degradation, uracil => beta-alanine, thymine => 3-aminoisobutanoate | 2 | 0.06 |
| **M00746** | Multidrug resistance, repression of porin OmpF | 1 | 0.03 |
| **M00027** | GABA (gamma-Aminobutyrate) shunt | 1 | 0.03 |

Supplementary Table S4. *Enterobacter spp.* KEGG-pathways.

| KEGG-pathway ID | KEGG-pathway description | Genomes | Frequency |
|---|---|---|---|
| **00010** | Glycolysis / Gluconeogenesis | 35 | 1 |
| **00020** | Citrate cycle (TCA cycle) | 35 | 1 |
| **00030** | Pentose phosphate pathway | 35 | 1 |
| **00040** | Pentose and glucuronate interconversions | 35 | 1 |
| **00051** | Fructose and mannose metabolism | 35 | 1 |
| **00052** | Galactose metabolism | 35 | 1 |
| **00053** | Ascorbate and aldarate metabolism | 35 | 1 |
| **00061** | Fatty acid biosynthesis | 35 | 1 |
| **00071** | Fatty acid degradation | 35 | 1 |
| **00130** | Ubiquinone and other terpenoid-quinone biosynthesis | 35 | 1 |
| **00190** | Oxidative phosphorylation | 35 | 1 |
| **00220** | Arginine biosynthesis | 35 | 1 |
| **00230** | Purine metabolism | 35 | 1 |
| **00240** | Pyrimidine metabolism | 35 | 1 |
| **00250** | Alanine, aspartate and glutamate metabolism | 35 | 1 |
| **00260** | Glycine, serine and threonine metabolism | 35 | 1 |
| **00261** | Monobactam biosynthesis | 35 | 1 |
| **00270** | Cysteine and methionine metabolism | 35 | 1 |
| **00280** | Valine, leucine and isoleucine degradation | 35 | 1 |
| **00281** | Geraniol degradation | 35 | 1 |
| **00290** | Valine, leucine and isoleucine biosynthesis | 35 | 1 |
| **00300** | Lysine biosynthesis | 35 | 1 |
| **00310** | Lysine degradation | 35 | 1 |
| **00330** | Arginine and proline metabolism | 35 | 1 |
| **00332** | Carbapenem biosynthesis | 35 | 1 |
| **00340** | Histidine metabolism | 35 | 1 |
| **00350** | Tyrosine metabolism | 35 | 1 |
| **00360** | Phenylalanine metabolism | 35 | 1 |
| **00362** | Benzoate degradation | 35 | 1 |
| **00380** | Tryptophan metabolism | 35 | 1 |
| **00400** | Phenylalanine, tyrosine and tryptophan biosynthesis | 35 | 1 |
| **00401** | Novobiocin biosynthesis | 35 | 1 |
| **00410** | beta-Alanine metabolism | 35 | 1 |
| **00430** | Taurine and hypotaurine metabolism | 35 | 1 |
| **00440** | Phosphonate and phosphinate metabolism | 35 | 1 |
| **00450** | Selenocompound metabolism | 35 | 1 |
| **00460** | Cyanoamino acid metabolism | 35 | 1 |
| **00471** | D-Glutamine and D-glutamate metabolism | 35 | 1 |
| **00473** | D-Alanine metabolism | 35 | 1 |
| **00480** | Glutathione metabolism | 35 | 1 |

Supplementary Table S4 (continuation). *Enterobacter spp.* KEGG-pathways.

| **KEGG-pathway ID** | **KEGG-pathway description** | **Genomes** | **Frequency** |
|---|---|---|---|
| **00500** | Starch and sucrose metabolism | 35 | 1 |
| **00511** | Other glycan degradation | 35 | 1 |
| **00520** | Amino sugar and nucleotide sugar metabolism | 35 | 1 |
| **00521** | Streptomycin biosynthesis | 35 | 1 |
| **00523** | Polyketide sugar unit biosynthesis | 35 | 1 |
| **00540** | Lipopolysaccharide biosynthesis | 35 | 1 |
| **00550** | Peptidoglycan biosynthesis | 35 | 1 |
| **00561** | Glycerolipid metabolism | 35 | 1 |
| **00562** | Inositol phosphate metabolism | 35 | 1 |
| **00564** | Glycerophospholipid metabolism | 35 | 1 |
| **00565** | Ether lipid metabolism | 35 | 1 |
| **00590** | Arachidonic acid metabolism | 35 | 1 |
| **00592** | alpha-Linolenic acid metabolism | 35 | 1 |
| **00620** | Pyruvate metabolism | 35 | 1 |
| **00625** | Chloroalkane and chloroalkene degradation | 35 | 1 |
| **00626** | Naphthalene degradation | 35 | 1 |
| **00627** | Aminobenzoate degradation | 35 | 1 |
| **00630** | Glyoxylate and dicarboxylate metabolism | 35 | 1 |
| **00633** | Nitrotoluene degradation | 35 | 1 |
| **00640** | Propanoate metabolism | 35 | 1 |
| **00650** | Butanoate metabolism | 35 | 1 |
| **00660** | C5-Branched dibasic acid metabolism | 35 | 1 |
| **00670** | One carbon pool by folate | 35 | 1 |
| **00680** | Methane metabolism | 35 | 1 |
| **00730** | Thiamine metabolism | 35 | 1 |
| **00740** | Riboflavin metabolism | 35 | 1 |
| **00750** | Vitamin B6 metabolism | 35 | 1 |
| **00760** | Nicotinate and nicotinamide metabolism | 35 | 1 |
| **00770** | Pantothenate and CoA biosynthesis | 35 | 1 |
| **00780** | Biotin metabolism | 35 | 1 |
| **00785** | Lipoic acid metabolism | 35 | 1 |
| **00790** | Folate biosynthesis | 35 | 1 |
| **00860** | Porphyrin and chlorophyll metabolism | 35 | 1 |
| **00900** | Terpenoid backbone biosynthesis | 35 | 1 |
| **00903** | Limonene and pinene degradation | 35 | 1 |
| **00910** | Nitrogen metabolism | 35 | 1 |
| **00920** | Sulfur metabolism | 35 | 1 |
| **00930** | Caprolactam degradation | 35 | 1 |
| **00970** | Aminoacyl-tRNA biosynthesis | 35 | 1 |
| **01040** | Biosynthesis of unsaturated fatty acids | 35 | 1 |

Supplementary Table S4 (continuation). *Enterobacter spp.* KEGG-pathways.

| KEGG-pathway ID | KEGG-pathway description | Genomes | Frequency |
|---|---|---|---|
| **01053** | Biosynthesis of siderophore group nonribosomal peptides | 35 | 1 |
| **01100** | Metabolic pathways | 35 | 1 |
| **01110** | Biosynthesis of secondary metabolites | 35 | 1 |
| **01120** | Microbial metabolism in diverse environments | 35 | 1 |
| **01130** | Biosynthesis of antibiotics | 35 | 1 |
| **01200** | Carbon metabolism | 35 | 1 |
| **01210** | 2-Oxocarboxylic acid metabolism | 35 | 1 |
| **01212** | Fatty acid metabolism | 35 | 1 |
| **01220** | Degradation of aromatic compounds | 35 | 1 |
| **01230** | Biosynthesis of amino acids | 35 | 1 |
| **01501** | beta-Lactam resistance | 35 | 1 |
| **01502** | Vancomycin resistance | 35 | 1 |
| **01503** | Cationic antimicrobial peptide (CAMP) resistance | 35 | 1 |
| **02010** | ABC transporters | 35 | 1 |
| **02020** | Two-component system | 35 | 1 |
| **02024** | Quorum sensing | 35 | 1 |
| **02030** | Bacterial chemotaxis | 35 | 1 |
| **02060** | Phosphotransferase system (PTS) | 35 | 1 |
| **03010** | Ribosome | 35 | 1 |
| **03018** | RNA degradation | 35 | 1 |
| **03020** | RNA polymerase | 35 | 1 |
| **03030** | DNA replication | 35 | 1 |
| **03060** | Protein export | 35 | 1 |
| **03070** | Bacterial secretion system | 35 | 1 |
| **03410** | Base excision repair | 35 | 1 |
| **03420** | Nucleotide excision repair | 35 | 1 |
| **03430** | Mismatch repair | 35 | 1 |
| **03440** | Homologous recombination | 35 | 1 |
| **04122** | Sulfur relay system | 35 | 1 |
| **00361** | Chlorocyclohexane and chlorobenzene degradation | 34 | 0.97 |
| **00364** | Fluorobenzoate degradation | 34 | 0.97 |
| **00623** | Toluene degradation | 34 | 0.97 |
| **02040** | Flagellar assembly | 34 | 0.97 |
| **00525** | Acarbose and validamycin biosynthesis | 32 | 0.91 |
| **00600** | Sphingolipid metabolism | 25 | 0.71 |
| **00072** | Synthesis and degradation of ketone bodies | 18 | 0.51 |
| **00791** | Atrazine degradation | 12 | 0.34 |
| **00405** | Phenazine biosynthesis | 10 | 0.29 |
| **00643** | Styrene degradation | 9 | 0.26 |
| **01054** | Nonribosomal peptide structures | 5 | 0.14 |

Supplementary Table S4 (continuation). *Enterobacter spp.* KEGG-pathways.

| KEGG-pathway ID | KEGG-pathway description | Genomes | Frequency |
|---|---|---|---|
| **00622** | Xylene degradation | 5 | 0.14 |
| **00621** | Dioxin degradation | 3 | 0.09 |
| **00591** | Linoleic acid metabolism | 2 | 0.06 |
| **00472** | D-Arginine and D-ornithine metabolism | 2 | 0.06 |
| **00121** | Secondary bile acid biosynthesis | 1 | 0.03 |

Supplementary Table S5. *P. aeruginosa KEGG-modules*

| KEGG-module ID | KEGG-module description | Genomes | Frequency |
|---|---|---|---|
| **M00002** | Glycolysis, core module involving three-carbon compounds | 21 | 1 |
| **M00007** | Pentose phosphate pathway, non-oxidative phase, fructose 6P => ribose 5P | 21 | 1 |
| **M00008** | Entner-Doudoroff pathway, glucose-6P => glyceraldehyde-3P + pyruvate | 21 | 1 |
| **M00009** | Citrate cycle (TCA cycle, Krebs cycle) | 21 | 1 |
| **M00010** | Citrate cycle, first carbon oxidation, oxaloacetate => 2-oxoglutarate | 21 | 1 |
| **M00011** | Citrate cycle, second carbon oxidation, 2-oxoglutarate => oxaloacetate | 21 | 1 |
| **M00015** | Proline biosynthesis, glutamate => proline | 21 | 1 |
| **M00016** | Lysine biosynthesis, succinyl-DAP pathway, aspartate => lysine | 21 | 1 |
| **M00018** | Threonine biosynthesis, aspartate => homoserine => threonine | 21 | 1 |
| **M00019** | Valine/isoleucine biosynthesis, pyruvate => valine / 2-oxobutanoate => isoleucine | 21 | 1 |
| **M00020** | Serine biosynthesis, glycerate-3P => serine | 21 | 1 |
| **M00021** | Cysteine biosynthesis, serine => cysteine | 21 | 1 |
| **M00023** | Tryptophan biosynthesis, chorismate => tryptophan | 21 | 1 |
| **M00024** | Phenylalanine biosynthesis, chorismate => phenylalanine | 21 | 1 |
| **M00028** | Ornithine biosynthesis, glutamate => ornithine | 21 | 1 |
| **M00045** | Histidine degradation, histidine => N-formiminoglutamate => glutamate | 21 | 1 |
| **M00046** | Pyrimidine degradation, uracil => beta-alanine, thymine => 3-aminoisobutanoate | 21 | 1 |
| **M00048** | Inosine monophosphate biosynthesis, PRPP + glutamine => IMP | 21 | 1 |
| **M00049** | Adenine ribonucleotide biosynthesis, IMP => ADP,ATP | 21 | 1 |
| **M00050** | Guanine ribonucleotide biosynthesis IMP => GDP,GTP | 21 | 1 |
| **M00052** | Pyrimidine ribonucleotide biosynthesis, UMP => UDP/UTP,CDP/CTP | 21 | 1 |
| **M00053** | Pyrimidine deoxyribonuleotide biosynthesis, CDP/CTP => dCDP/dCTP,dTDP/dTTP | 21 | 1 |
| **M00063** | CMP-KDO biosynthesis | 21 | 1 |
| **M00064** | ADP-L-glycero-D-manno-heptose biosynthesis | 21 | 1 |
| **M00082** | Fatty acid biosynthesis, initiation | 21 | 1 |
| **M00083** | Fatty acid biosynthesis, elongation | 21 | 1 |
| **M00086** | beta-Oxidation, acyl-CoA synthesis | 21 | 1 |
| **M00087** | beta-Oxidation | 21 | 1 |

Supplementary Table S5 (continuation). *P. aeruginosa KEGG-modules*

| KEGG-module ID | KEGG-module description | Genomes | Frequency |
|---|---|---|---|
| **M00093** | Phosphatidylethanolamine (PE) biosynthesis, PA => PS => PE | 21 | 1 |
| **M00115** | NAD biosynthesis, aspartate => NAD | 21 | 1 |
| **M00118** | Glutathione biosynthesis, glutamate => glutathione | 21 | 1 |
| **M00119** | Pantothenate biosynthesis, valine/L-aspartate => pantothenate | 21 | 1 |
| **M00120** | Coenzyme A biosynthesis, pantothenate => CoA | 21 | 1 |
| **M00122** | Cobalamin biosynthesis, cobinamide => cobalamin | 21 | 1 |
| **M00123** | Biotin biosynthesis, pimeloyl-ACP/CoA => biotin | 21 | 1 |
| **M00124** | Pyridoxal biosynthesis, erythrose-4P => pyridoxal-5P | 21 | 1 |
| **M00125** | Riboflavin biosynthesis, GTP => riboflavin/FMN/FAD | 21 | 1 |
| **M00126** | Tetrahydrofolate biosynthesis, GTP => THF | 21 | 1 |
| **M00135** | GABA biosynthesis, eukaryotes, putrescine => GABA | 21 | 1 |
| **M00144** | NADH:quinone oxidoreductase, prokaryotes | 21 | 1 |
| **M00149** | Succinate dehydrogenase, prokaryotes | 21 | 1 |
| **M00151** | Cytochrome bc1 complex respiratory unit | 21 | 1 |
| **M00155** | Cytochrome c oxidase, prokaryotes | 21 | 1 |
| **M00157** | F-type ATPase, prokaryotes and chloroplasts | 21 | 1 |
| **M00176** | Assimilatory sulfate reduction, sulfate => H2S | 21 | 1 |
| **M00307** | Pyruvate oxidation, pyruvate => acetyl-CoA | 21 | 1 |
| **M00338** | Cysteine biosynthesis, homocysteine + serine => cysteine | 21 | 1 |
| **M00417** | Cytochrome o ubiquinol oxidase | 21 | 1 |
| **M00432** | Leucine biosynthesis, 2-oxoisovalerate => 2-oxoisocaproate | 21 | 1 |
| **M00529** | Denitrification, nitrate => nitrogen | 21 | 1 |
| **M00551** | Benzoate degradation, benzoate => catechol / methylbenzoate => methylcatechol | 21 | 1 |
| **M00555** | Betaine biosynthesis, choline => betaine | 21 | 1 |
| **M00568** | Catechol ortho-cleavage, catechol => 3-oxoadipate | 21 | 1 |
| **M00570** | Isoleucine biosynthesis, threonine => 2-oxobutanoate => isoleucine | 21 | 1 |
| **M00572** | Pimeloyl-ACP biosynthesis, BioC-BioH pathway, malonyl-ACP => pimeloyl-ACP | 21 | 1 |
| **M00579** | Phosphate acetyltransferase-acetate kinase pathway, acetyl-CoA => acetate | 21 | 1 |
| **M00616** | Sulfate-sulfur assimilation | 21 | 1 |
| **M00637** | Anthranilate degradation, anthranilate => catechol | 21 | 1 |
| **M00639** | Multidrug resistance, efflux pump MexCD-OprJ | 21 | 1 |
| **M00642** | Multidrug resistance, efflux pump MexJK-OprM | 21 | 1 |
| **M00761** | Undecaprenylphosphate alpha-L-Ara4N biosynthesis, UDP-GlcA => undecaprenyl phosphate alpha-L-Ara4N | 21 | 1 |

Supplementary Table S5 (continuation). *P. aeruginosa KEGG-modules*

| KEGG-module ID | KEGG-module description | Genomes | Frequency |
|---|---|---|---|
| **M00793** | dTDP-L-rhamnose biosynthesis | 21 | 1 |
| **M00844** | Arginine biosynthesis, ornithine => arginine | 21 | 1 |
| **M00846** | Siroheme biosynthesis, glutamate => siroheme | 21 | 1 |
| **M00003** | Gluconeogenesis, oxaloacetate => fructose-6P | 20 | 0.95 |
| **M00005** | PRPP biosynthesis, ribose 5P => PRPP | 20 | 0.95 |
| **M00036** | Leucine degradation, leucine => acetoacetate + acetyl-CoA | 20 | 0.95 |
| **M00530** | Dissimilatory nitrate reduction, nitrate => ammonia | 20 | 0.95 |
| **M00533** | Homoprotocatechuate degradation, homoprotocatechuate => 2-oxohept-3-enedioate | 20 | 0.95 |
| **M00718** | Multidrug resistance, efflux pump MexAB-OprM | 20 | 0.95 |
| **M00745** | Imipenem resistance, repression of porin OprD | 20 | 0.95 |
| **M00835** | Pyocyanine biosynthesis, chorismate => pyocyanine | 20 | 0.95 |
| **M00156** | Cytochrome c oxidase, cbb3-type | 19 | 0.90 |
| **M00022** | Shikimate pathway, phosphoenolpyruvate + erythrose-4P => chorismate | 18 | 0.86 |
| **M00641** | Multidrug resistance, efflux pump MexEF-OprN | 17 | 0.81 |
| **M00769** | Multidrug resistance, efflux pump MexPQ-OpmE | 17 | 0.81 |
| **M00643** | Multidrug resistance, efflux pump MexXY-OprM | 16 | 0.76 |
| **M00035** | Methionine degradation | 8 | 0.38 |
| **M00026** | Histidine biosynthesis, PRPP => histidine | 4 | 0.19 |
| **M00851** | Carbapenem resistance | 3 | 0.14 |

Supplementary Table S6. *P. aeruginosa KEGG-pathways*

| KEGG-pathway ID | KEGG-pathway description | Genomes | Frequency |
|---|---|---|---|
| **00010** | Glycolysis / Gluconeogenesis | 21 | 1 |
| **00020** | Citrate cycle (TCA cycle) | 21 | 1 |
| **00030** | Pentose phosphate pathway | 21 | 1 |
| **00040** | Pentose and glucuronate interconversions | 21 | 1 |
| **00051** | Fructose and mannose metabolism | 21 | 1 |
| **00052** | Galactose metabolism | 21 | 1 |
| **00053** | Ascorbate and aldarate metabolism | 21 | 1 |
| **00061** | Fatty acid biosynthesis | 21 | 1 |
| **00071** | Fatty acid degradation | 21 | 1 |
| **00130** | Ubiquinone and other terpenoid-quinone biosynthesis | 21 | 1 |
| **00190** | Oxidative phosphorylation | 21 | 1 |
| **00220** | Arginine biosynthesis | 21 | 1 |
| **00230** | Purine metabolism | 21 | 1 |
| **00240** | Pyrimidine metabolism | 21 | 1 |
| **00250** | Alanine, aspartate and glutamate metabolism | 21 | 1 |
| **00260** | Glycine, serine and threonine metabolism | 21 | 1 |
| **00261** | Monobactam biosynthesis | 21 | 1 |
| **00270** | Cysteine and methionine metabolism | 21 | 1 |
| **00280** | Valine, leucine and isoleucine degradation | 21 | 1 |
| **00281** | Geraniol degradation | 21 | 1 |
| **00290** | Valine, leucine and isoleucine biosynthesis | 21 | 1 |
| **00300** | Lysine biosynthesis | 21 | 1 |
| **00310** | Lysine degradation | 21 | 1 |
| **00330** | Arginine and proline metabolism | 21 | 1 |
| **00332** | Carbapenem biosynthesis | 21 | 1 |
| **00340** | Histidine metabolism | 21 | 1 |
| **00350** | Tyrosine metabolism | 21 | 1 |
| **00360** | Phenylalanine metabolism | 21 | 1 |
| **00361** | Chlorocyclohexane and chlorobenzene degradation | 21 | 1 |
| **00362** | Benzoate degradation | 21 | 1 |
| **00364** | Fluorobenzoate degradation | 21 | 1 |
| **00380** | Tryptophan metabolism | 21 | 1 |
| **00400** | Phenylalanine, tyrosine and tryptophan biosynthesis | 21 | 1 |
| **00401** | Novobiocin biosynthesis | 21 | 1 |
| **00405** | Phenazine biosynthesis | 21 | 1 |
| **00410** | beta-Alanine metabolism | 21 | 1 |
| **00430** | Taurine and hypotaurine metabolism | 21 | 1 |
| **00440** | Phosphonate and phosphinate metabolism | 21 | 1 |
| **00450** | Selenocompound metabolism | 21 | 1 |
| **00460** | Cyanoamino acid metabolism | 21 | 1 |

Supplementary Table S6 (continuation). *P. aeruginosa KEGG-pathways*

| KEGG-pathway ID | KEGG-pathway description | Genomes | Frequency |
|---|---|---|---|
| **00471** | D-Glutamine and D-glutamate metabolism | 21 | 1 |
| **00472** | D-Arginine and D-ornithine metabolism | 21 | 1 |
| **00473** | D-Alanine metabolism | 21 | 1 |
| **00480** | Glutathione metabolism | 21 | 1 |
| **00500** | Starch and sucrose metabolism | 21 | 1 |
| **00520** | Amino sugar and nucleotide sugar metabolism | 21 | 1 |
| **00521** | Streptomycin biosynthesis | 21 | 1 |
| **00523** | Polyketide sugar unit biosynthesis | 21 | 1 |
| **00525** | Acarbose and validamycin biosynthesis | 21 | 1 |
| **00540** | Lipopolysaccharide biosynthesis | 21 | 1 |
| **00550** | Peptidoglycan biosynthesis | 21 | 1 |
| **00561** | Glycerolipid metabolism | 21 | 1 |
| **00562** | Inositol phosphate metabolism | 21 | 1 |
| **00564** | Glycerophospholipid metabolism | 21 | 1 |
| **00565** | Ether lipid metabolism | 21 | 1 |
| **00590** | Arachidonic acid metabolism | 21 | 1 |
| **00592** | alpha-Linolenic acid metabolism | 21 | 1 |
| **00600** | Sphingolipid metabolism | 21 | 1 |
| **00620** | Pyruvate metabolism | 21 | 1 |
| **00622** | Xylene degradation | 21 | 1 |
| **00623** | Toluene degradation | 21 | 1 |
| **00625** | Chloroalkane and chloroalkene degradation | 21 | 1 |
| **00626** | Naphthalene degradation | 21 | 1 |
| **00627** | Aminobenzoate degradation | 21 | 1 |
| **00630** | Glyoxylate and dicarboxylate metabolism | 21 | 1 |
| **00633** | Nitrotoluene degradation | 21 | 1 |
| **00640** | Propanoate metabolism | 21 | 1 |
| **00643** | Styrene degradation | 21 | 1 |
| **00650** | Butanoate metabolism | 21 | 1 |
| **00660** | C5-Branched dibasic acid metabolism | 21 | 1 |
| **00670** | One carbon pool by folate | 21 | 1 |
| **00680** | Methane metabolism | 21 | 1 |
| **00730** | Thiamine metabolism | 21 | 1 |
| **00740** | Riboflavin metabolism | 21 | 1 |
| **00750** | Vitamin B6 metabolism | 21 | 1 |
| **00760** | Nicotinate and nicotinamide metabolism | 21 | 1 |
| **00770** | Pantothenate and CoA biosynthesis | 21 | 1 |
| **00780** | Biotin metabolism | 21 | 1 |
| **00785** | Lipoic acid metabolism | 21 | 1 |
| **00790** | Folate biosynthesis | 21 | 1 |

Supplementary Table S6 (continuation). *P. aeruginosa KEGG-pathways*

| KEGG-pathway ID | KEGG-pathway description | Genomes | Frequency |
|---|---|---|---|
| **00860** | Porphyrin and chlorophyll metabolism | 21 | 1 |
| **00900** | Terpenoid backbone biosynthesis | 21 | 1 |
| **00903** | Limonene and pinene degradation | 21 | 1 |
| **00910** | Nitrogen metabolism | 21 | 1 |
| **00920** | Sulfur metabolism | 21 | 1 |
| **00930** | Caprolactam degradation | 21 | 1 |
| **00970** | Aminoacyl-tRNA biosynthesis | 21 | 1 |
| **01040** | Biosynthesis of unsaturated fatty acids | 21 | 1 |
| **01053** | Biosynthesis of siderophore group nonribosomal peptides | 21 | 1 |
| **01100** | Metabolic pathways | 21 | 1 |
| **01110** | Biosynthesis of secondary metabolites | 21 | 1 |
| **01120** | Microbial metabolism in diverse environments | 21 | 1 |
| **01130** | Biosynthesis of antibiotics | 21 | 1 |
| **01200** | Carbon metabolism | 21 | 1 |
| **01210** | 2-Oxocarboxylic acid metabolism | 21 | 1 |
| **01212** | Fatty acid metabolism | 21 | 1 |
| **01220** | Degradation of aromatic compounds | 21 | 1 |
| **01230** | Biosynthesis of amino acids | 21 | 1 |
| **01501** | beta-Lactam resistance | 21 | 1 |
| **01502** | Vancomycin resistance | 21 | 1 |
| **01503** | Cationic antimicrobial peptide (CAMP) resistance | 21 | 1 |
| **02010** | ABC transporters | 21 | 1 |
| **02020** | Two-component system | 21 | 1 |
| **02024** | Quorum sensing | 21 | 1 |
| **02025** | Biofilm formation - Pseudomonas aeruginosa | 21 | 1 |
| **02030** | Bacterial chemotaxis | 21 | 1 |
| **02040** | Flagellar assembly | 21 | 1 |
| **02060** | Phosphotransferase system (PTS) | 21 | 1 |
| **03010** | Ribosome | 21 | 1 |
| **03018** | RNA degradation | 21 | 1 |
| **03020** | RNA polymerase | 21 | 1 |
| **03030** | DNA replication | 21 | 1 |
| **03060** | Protein export | 21 | 1 |
| **03070** | Bacterial secretion system | 21 | 1 |
| **03410** | Base excision repair | 21 | 1 |
| **03420** | Nucleotide excision repair | 21 | 1 |
| **03430** | Mismatch repair | 21 | 1 |
| **03440** | Homologous recombinatio | 21 | 1 |
| **03450** | Non-homologous end-joining | 21 | 1 |

Supplementary Table S6 (continuation). *P. aeruginosa KEGG-pathways*

| KEGG-pathway ID | KEGG-pathway description | Genomes | Frequency |
|---|---|---|---|
| **04122** | Sulfur relay system | 21 | 1 |
| **00072** | Synthesis and degradation of ketone bodies | 20 | 0.95 |

Supplementary Table S7. *Mycopalsma spp. KEGG-modules*

| KEGG-module ID | KEGG-module description | Genomes | Frequency |
|---|---|---|---|
| **M00005** | PRPP biosynthesis, ribose 5P => PRPP | 93 | 1 |
| **M00157** | F-type ATPase, prokaryotes and chloroplasts | 91 | 0.98 |
| **M00002** | Glycolysis, core module involving three-carbon compounds | 89 | 0.96 |
| **M00579** | Phosphate acetyltransferase-acetate kinase pathway, acetyl-CoA => acetate | 72 | 0.77 |
| **M00307** | Pyruvate oxidation, pyruvate => acetyl-CoA | 70 | 0.75 |
| **M00001** | Glycolysis (Embden-Meyerhof pathway), glucose => pyruvate | 31 | 0.33 |
| **M00049** | Adenine ribonucleotide biosynthesis, IMP => ADP,ATP | 23 | 0.25 |
| **M00050** | Guanine ribonucleotide biosynthesis IMP => GDP,GTP | 12 | 0.13 |
| **M00549** | Nucleotide sugar biosynthesis, glucose => UDP-glucose | 8 | 0.09 |
| **M00120** | Coenzyme A biosynthesis, pantothenate => CoA | 4 | 0.04 |
| **M00140** | C1-unit interconversion, prokaryotes | 1 | 0.01 |
| **M00854** | Glycogen biosynthesis, glucose-1P => glycogen/starch | 1 | 0.01 |
| **M00048** | Inosine monophosphate biosynthesis, PRPP + glutamine => IMP | 1 | 0.01 |

Supplementary Table S8. *Mycopalsma spp. KEGG-pathways*

| KEGG-pathway ID | KEGG-pathway description | Genomes | Frequency |
|---|---|---|---|
| **00010** | Glycolysis / Gluconeogenesis | 93 | 1 |
| **00030** | Pentose phosphate pathway | 93 | 1 |
| **00051** | Fructose and mannose metabolism | 93 | 1 |
| **00190** | Oxidative phosphorylation | 93 | 1 |
| **00230** | Purine metabolism | 93 | 1 |
| **00240** | Pyrimidine metabolism | 93 | 1 |
| **00270** | Cysteine and methionine metabolism | 93 | 1 |
| **00450** | Selenocompound metabolism | 93 | 1 |
| **00480** | Glutathione metabolism | 93 | 1 |
| **00564** | Glycerophospholipid metabolism | 93 | 1 |
| **00620** | Pyruvate metabolism | 93 | 1 |
| **00680** | Methane metabolism | 93 | 1 |
| **00760** | Nicotinate and nicotinamide metabolism | 93 | 1 |
| **00970** | Aminoacyl-tRNA biosynthesis | 93 | 1 |
| **01100** | Metabolic pathways | 93 | 1 |
| **01110** | Biosynthesis of secondary metabolites | 93 | 1 |
| **01120** | Microbial metabolism in diverse environments | 93 | 1 |
| **01130** | Biosynthesis of antibiotics | 93 | 1 |
| **01200** | Carbon metabolism | 93 | 1 |
| **01230** | Biosynthesis of amino acids | 93 | 1 |
| **02010** | ABC transporters | 93 | 1 |
| **02024** | Quorum sensing | 93 | 1 |
| **03010** | Ribosome | 93 | 1 |
| **03018** | RNA degradation | 93 | 1 |
| **03030** | DNA replication | 93 | 1 |
| **03060** | Protein export | 93 | 1 |
| **03070** | Bacterial secretion system | 93 | 1 |
| **03410** | Base excision repair | 93 | 1 |
| **03420** | Nucleotide excision repair | 93 | 1 |
| **03430** | Mismatch repair | 93 | 1 |
| **03440** | Homologous recombination | 93 | 1 |
| **03020** | RNA polymerase | 92 | 0.99 |
| **00500** | Starch and sucrose metabolism | 89 | 0.96 |
| **00260** | Glycine, serine and threonine metabolism | 86 | 0.92 |
| **00520** | Amino sugar and nucleotide sugar metabolism | 86 | 0.92 |
| **00640** | Propanoate metabolism | 84 | 0.90 |
| **00561** | Glycerolipid metabolism | 83 | 0.89 |
| **00730** | Thiamine metabolism | 83 | 0.89 |
| **02060** | Phosphotransferase system (PTS) | 83 | 0.89 |
| **00020** | Citrate cycle (TCA cycle) | 74 | 0.80 |

Supplementary Table S8 (contonuation). *Mycopalsma spp. KEGG-pathways*

| KEGG-pathway ID | KEGG-pathway description | Genomes | Frequency |
|---|---|---|---|
| **00630** | Glyoxylate and dicarboxylate metabolism | 73 | 0.78 |
| **00670** | One carbon pool by folate | 73 | 0.78 |
| **00280** | Valine, leucine and isoleucine degradation | 72 | 0.77 |
| **00430** | Taurine and hypotaurine metabolism | 72 | 0.77 |
| **00740** | Riboflavin metabolism | 70 | 0.75 |
| **00770** | Pantothenate and CoA biosynthesis | 65 | 0.70 |
| **00052** | Galactose metabolism | 62 | 0.67 |
| **00040** | Pentose and glucuronate interconversions | 56 | 0.60 |
| **02020** | Two-component system | 53 | 0.57 |
| **00460** | Cyanoamino acid metabolism | 52 | 0.56 |
| **04122** | Sulfur relay system | 51 | 0.55 |
| **00625** | Chloroalkane and chloroalkene degradation | 39 | 0.42 |
| **00790** | Folate biosynthesis | 38 | 0.41 |
| **00053** | Ascorbate and aldarate metabolism | 35 | 0.38 |
| **00250** | Alanine, aspartate and glutamate metabolism | 27 | 0.29 |
| **00330** | Arginine and proline metabolism | 27 | 0.29 |
| **00380** | Tryptophan metabolism | 26 | 0.28 |
| **00220** | Arginine biosynthesis | 25 | 0.27 |
| **00310** | Lysine degradation | 25 | 0.27 |
| **00626** | Naphthalene degradation | 21 | 0.23 |
| **01220** | Degradation of aromatic compounds | 21 | 0.23 |
| **00900** | Terpenoid backbone biosynthesis | 15 | 0.16 |
| **00521** | Streptomycin biosynthesis | 15 | 0.16 |
| **00660** | C5-Branched dibasic acid metabolism | 13 | 0.14 |
| **00511** | Other glycan degradation | 9 | 0.10 |
| **00071** | Fatty acid degradation | 8 | 0.09 |
| **00562** | Inositol phosphate metabolism | 7 | 0.08 |
| **00650** | Butanoate metabolism | 3 | 0.03 |
| **00400** | Phenylalanine, tyrosine and tryptophan biosynthesis | 2 | 0.02 |
| **00590** | Arachidonic acid metabolism | 1 | 0.01 |
| **00860** | Porphyrin and chlorophyll metabolism | 1 | 0.01 |
| **00600** | Sphingolipid metabolism | 1 | 0.01 |
| **00340** | Histidine metabolism | 1 | 0.01 |
